# Performance Optimization of Triangular Cantilever Beam Piezoelectric Energy Harvesters: Synergistic Design Research on Mass Block Structure Optimization and Negative Poisson’s Ratio Substrate

**DOI:** 10.3390/mi17010078

**Published:** 2026-01-07

**Authors:** Ruijie Ren, Binbin Li, Jun Liu, Yu Zhang, Gang Xu, Weijia Liu

**Affiliations:** 1 Mechanical College, Shanghai Dianji University, Shanghai 201306, China; 23600002020618@st.sdju.edu.cn (R.R.); liujun@sdju.edu.cn (J.L.); 2 Kaiserslautern Institute of Intelligent Manufacturing, Shanghai DianJi University, Shanghai 201306, China; 3 Electrical Engineering College, Shanghai Dianji University, Shanghai 201306, China; 23610001030603@st.sdju.edu.cn (Y.Z.); 23600001010202@st.sdju.edu.cn (G.X.); 23600001010421@st.sdju.edu.cn (W.L.)

**Keywords:** triangular cantilever beam piezoelectric energy harvester, structure optimization, mass block, Negative Poisson’s ratio structure, finite element simulation

## Abstract

The widespread adoption of low-power devices and microelectronic systems has intensified the need for efficient energy harvesting solutions. While cantilever-beam piezoelectric energy harvesters (PEHs) are popular for their simplicity, their performance is often limited by conventional mass block designs. This study addresses this by proposing a comprehensive structural optimization framework for a triangular cantilever PEH to significantly enhance its electromechanical conversion efficiency. The methodology involved a multi-stage approach: first, an embedded coupling design was introduced to connect the mass block and cantilever beam, improving space utilization and strain distribution. Subsequently, the mass block’s shape was optimized. Furthermore, a negative Poisson’s ratio (NPR) honeycomb structure was integrated into the cantilever beam substrate to induce biaxial strain in the piezoelectric layer. Finally, a variable-density mass block was implemented. The synergistic combination of all optimizations—embedded coupling, NPR substrate, and variable-density mass block—culminated in a total performance enhancement of 69.07% (17.76 V) in voltage output and a 44.34% (28.01 Hz) reduction in resonant frequency. Through experimental testing, the output performance of the prototype machine showed good consistency with the simulation results, successfully verifying the effectiveness of the structural optimization method proposed in this study. These findings conclusively show that strategic morphological reconfiguration of key components is highly effective in developing high-performance, low-frequency adaptive piezoelectric energy harvesting systems.

## 1. Introduction

The advent of the Internet of Things (IoT) and the proliferation of low-power devices have accelerated the widespread adoption of microelectronic systems and low-power sensors. These technologies are now extensively deployed in embedded systems, aerospace applications, and health monitoring domains [1,2,3,4,5,6]. Microelectromechanical systems (MEMS) encounter significant maintenance challenges due to miniaturized dimensions, large-scale deployment, and wide distribution. Piezoelectric energy harvesting technology provides an innovative solution through converting mechanical energy into electricity [7,8,9]. Vibration energy, traditionally discarded as a waste byproduct in conventional energy conversion processes, can now be efficiently harvested as clean electricity through the piezoelectric devices. This approach not only enables comprehensive utilization of ambient vibrations but also pioneers novel circular paradigms for wasted energy resources [10,11,12]. Such technology broadens the scope of renewable energy applications while establishing new pathways for developing high-efficiency, sustainable distributed power supply systems [13,14,15].

For piezoelectric energy harvesters, material selection, structural design, energy harvesting circuits, and novel configurations significantly influence device performance, serving as critical factors in enhancing energy efficiency, stability, and application scope [16,17]. Regarding material selection, Li Hui et al. [18] employed finite element analysis to systematically reveal the effects of substrate materials, piezoelectric material properties, mass block design, cantilever beam dimensional parameters, and load conditions on output performance. In structural design, E. L. Pradees et al. [19] investigated the influence of mass block size (rectangular), geometry, and positioning on rectangular cantilever beams. Abdelkefi [20] explored altered mass block distributions by connecting two unequally distributed masses via a metal plate, using simulation experiments to demonstrate the impact of such configurations on output performance and natural frequencies. Chen et al. [21] developed a multi-branched cantilever piezoelectric energy harvester, replacing conventional mass blocks with a branched-arm configuration to achieve flexible frequency response modulation. Simulations verified that asymmetric Y-shaped branched piezoelectric energy harvesters exhibit a higher number of resonant frequencies and greater output power compared to symmetric counterparts. This finding offers novel design principles for enhanced energy harvesting. Paquin [22] investigated the influence of variable-thickness cantilever beams on electromechanical performance, validating through finite element analysis how beam slope angles affect output characteristics. The results indicated a 3.6-fold enhancement in output power at optimized beam geometries. Qizhou Lid [23] proposed a gravity-shift-based piezoelectric energy harvester, which achieved a maximum voltage of 12.67 V and a power output of 1.553 mW under optimal variable combinations. Furthermore, Elgamal, M.A. et al. [24] developed a novel nonlinear piezoelectric energy harvester array, which demonstrated a 3.24-fold enhancement in output power compared with conventional designs.

Ryo Ichige et al. modified the substrate of cantilever beams using concave hexagonal structures with negative Poisson’s ratio. Experimental verification demonstrated a 48% reduction in the resonant frequency of the energy harvester and a 3.2-fold increase in output power [25]. Chen et al. advanced a methodology for augmenting the power output of piezoelectric energy harvesting systems through the implementation of gradient-grown structures. The integration of cantilever beams with gradient auxetic structures has been demonstrated to result in a more uniform strain distribution across the cantilever. Experimental validation demonstrated a 355% increase in power density compared to conventional energy harvesters [26].

Erturk, A. et al. designed a piezomagnetoelastic structure incorporating nonlinear magnetic buckling [27]. Through the implementation of verification using the electromechanical equations of the nonlinear system and theoretical simulations, it was determined that the piezomagnetoelastic generator achieves a 200% increase in open-circuit voltage amplitude, thereby indicating an 800% increase in power amplitude. Zhou et al. proposed and validated a broadband piezoelectric vibration energy harvester based on a magnetically constructed three-stable state trap [28]. A mathematical model was formulated using the energy equation, and experimental data were utilized to optimize the identification system parameters. This analysis resulted in the characterization of the nonlinear restoring force as a high order polynomial. The numerical simulation results obtained under harmonic excitation at 1–20 Hz align with experimental findings, thereby confirming the validity of the modeled system. In comparison with conventional bistable configurations, this tristable design exhibits a greater propensity to traverse potential wells, consequently attaining higher energy output across a more extensive frequency range. Zou, D. et al. developed a device capable of customizing nonlinear forces [29]. The apparatus under consideration consists of a pre-compressed spring, bearings, and specially designed raceways. Through the implementation of simulation and experimental validation, the feasibility of this method was demonstrated for hardening nonlinearity, softening nonlinearity, bistable, tristable, and piecewise nonlinear systems. This apparatus enables designers to ascertain the most suitable nonlinear forces for dynamic systems.

While cantilever-beam piezoelectric energy harvesters (PEHs) offer advantages in structural simplicity and fabrication convenience [13,30,31], their conventional homogeneous mass block designs face intrinsic limitations in energy conversion efficiency [32,33,34]. The present study proposes a parametric optimization framework for the mass block structure and cantilever substrate based on the standard PEH architecture. A three-dimensional multiphysics coupling model is employed to systematically elucidate the impact of structural optimization on electromechanical performance. Additionally, a comparison is made between the output characteristics of the conventional and optimized structures. The results demonstrate that the optimized energy harvester structure effectively improves the stress distribution within the piezoelectric elements, thereby enhancing the piezoelectric effect. This, in turn, results in a substantial improvement in the harvester’s output performance and an increase in electromechanical conversion efficiency.

## 2. Energy Harvester Structure

**Figure 1:**
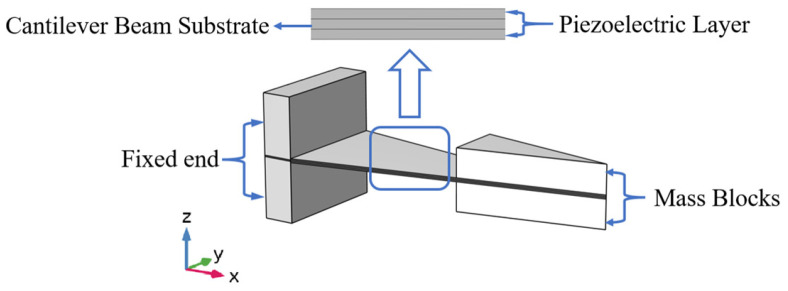
Structure Diagram of the Triangular Cantilever Beam Piezoelectric Energy Harvester.

The piezoelectric energy harvester operates via the direct piezoelectric effect to achieve mechanical-to-electrical energy conversion, where crystal deformation generates interfacial potential differences [35,36]. In cantilever beam systems, the structural parameters of the mass block significantly affect dynamics and electromechanical efficiency, with conventional designs exhibiting limited energy capture capability under broadband excitation. This study conducts systematic parameter optimization of mass blocks in triangular beam piezoelectric energy harvester, elucidating morphological regulation mechanisms on electromechanical responses, by constructing a nonlinear correlation model between geometrical parameters and the system’s intrinsic frequency/output voltage. As shown in Figure 1, the traditional triangular beam piezoelectric energy harvester is as follows:

The parametric design of key energy harvester components is parametrized as follows:

Piezoelectric Layer: A triangular PZT-5A piezoelectric ceramic with dimensions of 20 mm (width) × 50 mm (length) × 0.2 mm (height);

Cantilever Beam Substrate: Pure copper cantilever beam dimensionally matches to the piezoelectric layer (20 mm × 50 mm × 0.2 mm), featuring a clamped end region (20 mm × 5 mm).

The material parameters are delineated in Table 1.

In the Select Physical Fields tree select Structural Mechanics > Electromagnetic-Structural Interaction > Piezoelectric > Piezoelectric, Solid and select AC/DC > Circuits in the Select Physical Fields tree.

Boundary Constraints: Fully constrained displacement boundary conditions (u_x = u_y = u_z = 0) are applied to the left-side fixed end metal block.

Load Application: A vertical acceleration of 1 m/s^2^ is equivalently converted to a dis-tributed volume force acting on the entire system.

Electromechanical Coupling: Ideal electrode boundary conditions are defined on the top/bottom surfaces of the piezoelectric ceramic layer, with an external 12 kΩ load resistor connected to form a closed electrical circuit.

**Figure 2:**
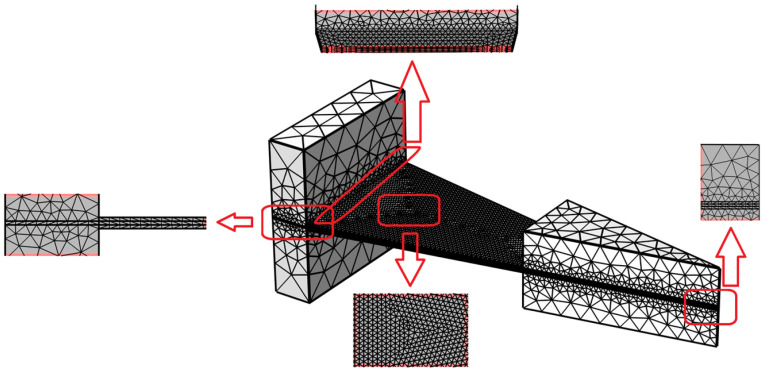
Finite Element Mesh Model of the Energy Harvester.

Mesh settings: Select “Roughened mesh for self-use,” ensuring a cell size ranging from 0.1 to 15 mm. The mesh’s coherence is attributed to the strategic decision to form a federation (fin) during the modeling process, a crucial aspect that enhances the mesh’s structural integrity and precision. The grid details are illustrated in the Figure 2.

This framework establishes a complete electromechanical energy conversion path- way, enabling concurrent multiphysics coupled-field analysis across mechanical deformation, piezoelectric polarization, and circuit response domains.

## 3. Finite Element Simulation and Output Response Analysis

The triangular cantilever piezoelectric energy harvester investigated in this study demonstrates three significant advantages over conventional rectangular configurations: Firstly, its tapered geometric configuration enables superior mechanical stress distribution characteristics. Secondly, the equivalent stiffness coefficient of the triangular structure decreases the first-order fundamental natural frequency of the system compared to the rectangular configuration, thus broadening the capture bandwidth for ambient vibrational energy. Thirdly, the triangular cantilever beam configuration offers a lighter mass while maintaining equivalent output power. Furthermore, the distinctive geometry of triangular cantilever beams provides enhanced design flexibility for the structure optimization of the mass block, enabling improved space utilization while maintaining structural dynamic stability.

### 3.1. Structure Optimization of Mass Blocks

#### 3.1.1. Research on Distribution Patterns of Mass Blocks

**Figure 3:**
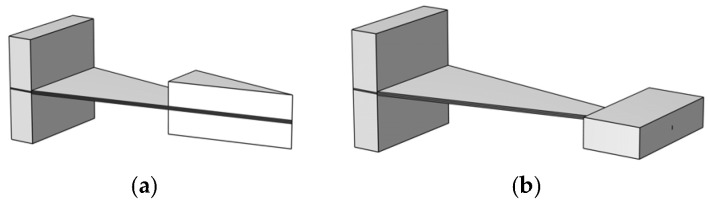
Schematic diagram of quality block structure optimization. (**a**) Structure Ⅰ; (**b**) Structure Ⅱ.

This study utilizes an embedded coupling method to connect the mass block with the cantilever beam, thereby addressing the prevalent issue of inadequate spatial utilization in the end region of conventional triangular cantilever beams. As demonstrated in Figure 3b, this composite structure establishes a geometrically matched cavity within the core region of the mass block. The free end of the triangular cantilever beam is embedded into this cavity, thereby creating an embedded coupling system between the mass block and the cantilever beam. Furthermore, the mass block of Structure Ⅱ undergoes a transformation from a triangular to a rectangular configuration. The two mass blocks possess equal weight and identical material properties, exhibiting a symmetrical configuration about the centerline of the base plate. The apex of the triangular cantilever beam in Structure Ⅱ is located at the center of the mass block’s cross-section.

Structure Ⅰ (Figure 3a) is a conventional cantilever beam piezoelectric energy harvester. The structure is uncomplicated, but the mass block’s space utilization is limited. The elongated mass block imposes constraints on the free vibration length of the cantilever beam, thereby reducing the volume of piezoelectric material available for deformation and constraining output performance. Structure II (Figure 3b) employs an embedded coupling design, with the cantilever beam end embedded into a slot in the mass block, forming a compact T-shaped structure. The mass block has undergone a transformation from a triangular to a rectangular configuration, thereby fully leveraging the spatial potential at the free end of the triangular cantilever beam. These two modifications result in substantial alterations to the system’s mass distribution and moment of inertia. A comparison of the two structures reveals that Structure Ⅱ effectively addresses the issue of insufficient space utilization. This approach ensures that a greater number of segments within the cantilever beam engage in vibrational deformation, thereby augmenting the effective volume of piezoelectric material. Consequently, this results in an increase in charge accumulation and an enhancement in output voltage.

**Figure 4:**
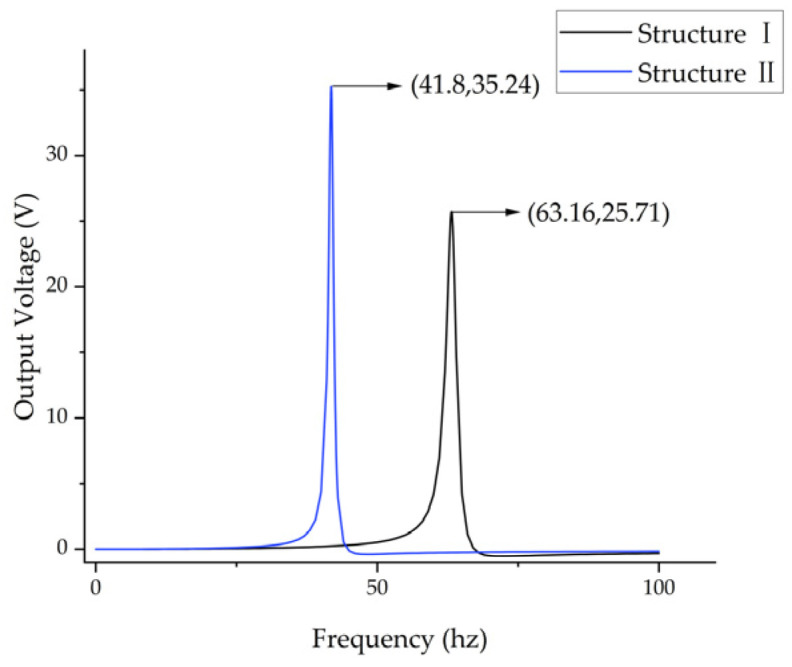
Finite element simulation results of the distribution pattern of the mass block.

**Figure 5:**
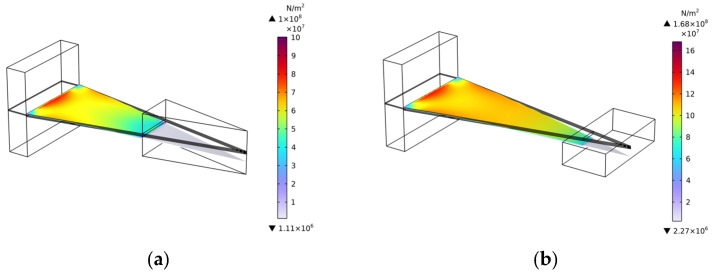
Piezoelectric sheet stress distribution diagram. (**a**) Structure Ⅰ; (**b**) Structure Ⅱ.

As illustrated in Figure 4, the output voltage-frequency curves for the two structures are presented, with the horizontal axis representing frequency (0–100 Hz) and the vertical axis representing output voltage. The black curve signifies the output curve of Structure Ⅰ, attaining a maximum output of 25.71 V at 63.16 Hz. The blue curve in the figure illustrates the output curve of Structure Ⅱ, which achieves a maximum output of 35.24 V at a frequency of 41.8 Hz. The findings of the study indicate that the embedded coupling connection method not only reduces the first-order natural frequency by 21.36 Hz (34.81%) but also increases the maximum output voltage by 9.53 V (37.06%), effectively optimizing the output performance of the energy harvester. Figure 5 presents the stress contour map of the piezoelectric element. A thorough examination of Figure 5 reveals two significant optimizations in the piezoelectric element of Structure Ⅱ. Firstly, the effective deformation volume of the piezoelectric element has increased. Secondly, the stress distribution across the piezoelectric element is more uniform, resulting in greater overall force absorption. The piezoelectric element generates an increased charge through their mutual interaction, thereby increasing the output voltage. The embedded coupling connection method has been demonstrated to adjust the bending stiffness and optimize the uniformity of bending strain distribution, thereby further enhancing energy harvesting efficiency.

#### 3.1.2. Topology Optimization of Mass Block Shapes

**Figure 6:**
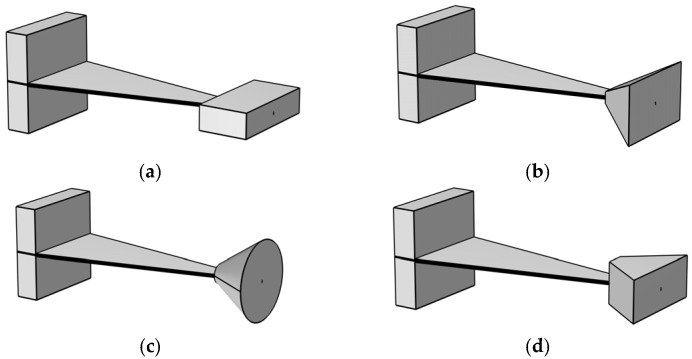
Schematic Structure Diagrams of Mass Block Structures with Various Shapes. (**a**) Structure Ⅰ; (**b**) Structure Ⅱ; (**c**) Structure Ⅲ; (**d**) Structure Ⅳ.

Building upon the previously described embedded coupling connection method, the structural shape of the mass block is modified to achieve the goal of optimizing the output performance of the energy harvester, as shown in Figure 6.

The four structures differ only in the shape of the mass block, while all other structural parameters remain constant. In the design of the mass block structure, it is imperative that the mass block’s weight remains constant, while the contact area and shape between the mass block and the cantilever beam must also remain constant. Among these, the widest end of the four-mass block structure measures 20 mm, maintaining the same length as the base of the triangular cantilever beam. The longitudinal length—that is, the length in the X direction—of all four masses is 10 mm.

In this study, the effects of four different mass block structures on the performance of the captive energy harvester in Figure 6 are analyzed comparatively.

**Figure 7:**
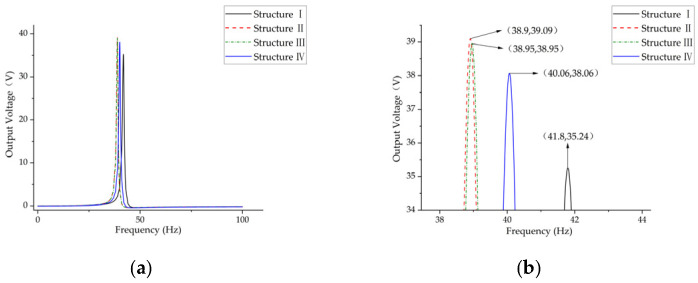
Frequency-Output Voltage Response Curve of Mass Block Structures with Various Shapes. (**a**) Full-band frequency spectrum; (**b**) Curve peak.

As demonstrated in Figure 7, the optimized structures exhibit superior output performance in comparison to structure I. The voltage and first-order natural frequency of structure I are recorded as 35.24 V and 41.8 Hz. Structure III demonstrates the least enhancement in performance, exhibiting an 8% rise in voltage (from 35.24 V to 38.06 V) and a 4.1% decline in natural frequency. The output curves of structures II (39.09 V, 38.9 Hz) and structures III (38.95 V, 38.9 Hz) exhibit a significant degree of overlap; however, it is evident that structure II possesses a higher output voltage and a lower intrinsic frequency. The findings of the present study demonstrate that, while maintaining constant parameters such as volume, contact area, and width, Structure II exhibits an augmentation in output voltage of 10.9% (35.24 V to 39.09 V) and a reduction in first-order natural frequency of 6.9% (38.9 Hz to 41.8 Hz) in comparison to Structure I.

**Figure 8:**
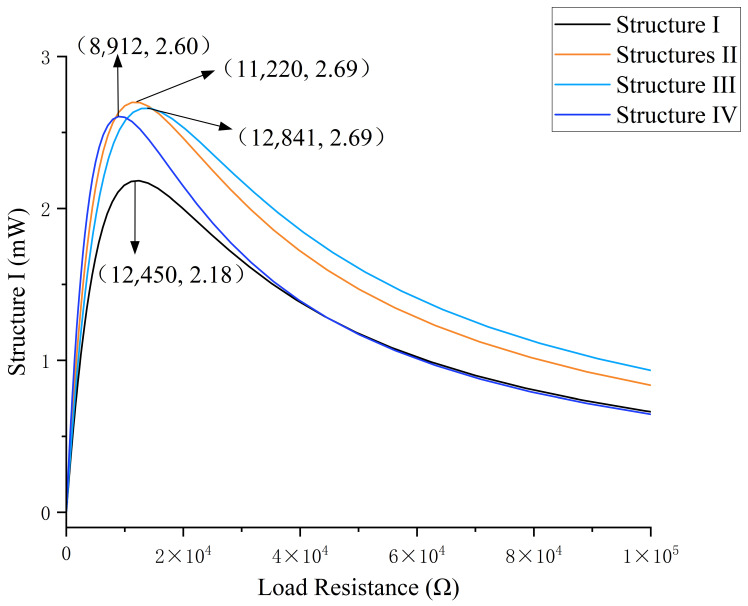
Power–Resistance load simulation response curve.

As demonstrated in Figure 8, the variation in output power of four distinct piezoelectric energy harvesters (Structures I–IV) is depicted as a function of load resistance. All structures manifest a distinctive single-peak curve characteristic, whereby output power escalates swiftly as load resistance ascends, attains a maximum value (peak power), and subsequently declines gradually as resistance persists in its increase. Structure Ⅰ demonstrated the poorest performance, achieving a peak power of 2.18 mW at 12,450 Ω. Conversely, Structure Ⅱ exhibited the optimal performance, reaching a peak power of 2.69 mW at 11,220 Ω. Structure Ⅲ demonstrates a reduced peak power output in comparison to Structure Ⅱ. However, beyond 20,000 Ω, Structure Ⅲ exhibits a marginal increase in power output relative to Structure Ⅱ. At maximum power output, the load resistance experienced by Structure Ⅳ is minimal. However, as the load increases, the rate of power decay accelerates rapidly. Beyond 35,000 Ω, the curve essentially overlaps with that of Structure Ⅰ.

**Figure 9:**
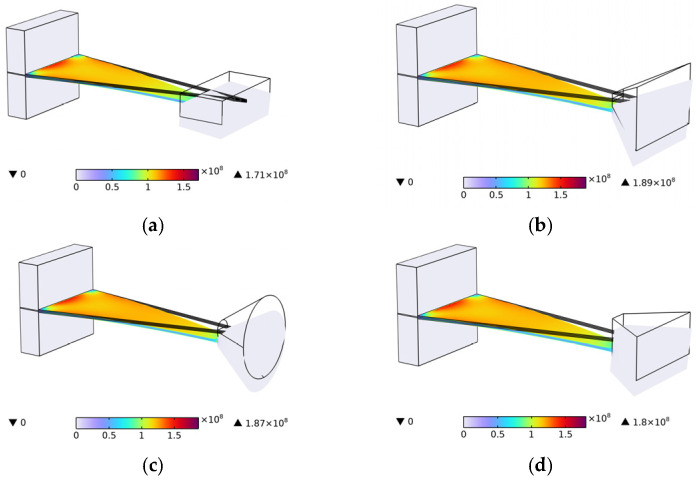
Stress Distribution at First-Order Natural Frequency of Mass Block Structures with Various Shapes. (**a**) Structure I; (**b**) Structure II; (**c**) Structure III; (**d**) Structure IV.

As illustrated in Figure 9, the stress distribution of a cantilever beam at its first natural frequency is depicted, with units expressed in N/m^2^. The structures of the four mass blocks differ in shape, resulting in varying positions of their centers of gravity. A comparison of the centers of gravity of the structures under consideration reveals that, in contrast to Structure Ⅰ, those of the remaining structures are situated in closer proximity to the free end. Consequently, these three structures experience elevated levels of stress during deformation and generate higher output voltages. A comparison of Structures Ⅱ and Ⅲ with Structure Ⅳ reveals a more pronounced trend of narrower front and wider rear mass blocks. Consequently, Structures Ⅱ and Ⅲ demonstrate superior output performance in comparison to Structure Ⅳ.

By calculating the center of gravity position of the mass blocks, the output voltage sensitivity coefficients of Structure Ⅱ and Structure Ⅲ can be obtained as 2.505 V/mm and 2.03 V/mm, respectively, from which it can be observed that Structure Ⅱ is a more superior structure. A comparative analysis reveals that structures with width differences (Structures II–IV) will effectively extend the force arm lengths and generate larger bending strains under the same excitation. It is important to note that, while achieving performance optimization, the stress distribution curves and maximum stress values of Structure II remain comparable to those of Structure I. This demonstrates that Structure II does not impose an additional stress burden on the cantilever beams. Furthermore, this morphological reconfiguration does not introduce the risk of localized stress concentration, which contributes to prolonging the service life of the cantilever beams.

### 3.2. Study on the Negative Poisson’s Ratio Cantilever Beam Substrate

Negative Poisson’s ratio structures constitute a substantial category of mechanical metamaterials, exhibiting notable advantages in energy harvesting as a result of their distinctive counterintuitive deformation behavior—namely, lateral contraction under compression and lateral expansion under tension. Building on the optimizations from the previous section, this section introduces a negative Poisson’s ratio structure combined with a cantilever beam substrate. This addresses the issue of uneven strain distribution in conventional cantilever beams.

In comparison to conventional flat-plate structures, the honeycomb structure (1) demonstrates a negative Poisson’s ratio, thereby facilitating the simultaneous stretching of the piezoelectric material in two directions. This enhancement in power generation in the d32 mode results in an overall increase in output power. (2) The structural stiffness of systems characterized by a negative Poisson’s ratio is found to be lower in comparison to that of flat plate structures possessing equivalent thickness. This phenomenon, consequently, results in the occurrence of stress concentration on the piezoelectric elements. This concentration effect contributes to enhancing the output characteristics of the energy harvester.

All simulation conditions are consistent with those outlined in Section 3.1. As demonstrated in Figure 9, both cantilever beam substrates possess identical dimensions, including length, width, and thickness. Structure a is characterized by a flat plate configuration, while structure b exhibits a honeycomb-like structure.

**Figure 10:**
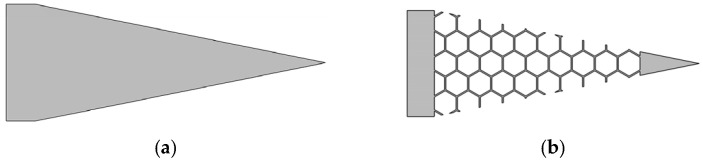
Schematic diagram of the cantilever beam base structure. (**a**) Top view of the Flat plate structure; (**b**) Top view of the Honeycomb structure.

A honeycomb structure with a negative Poisson ratio is composed of regular hexagonal cells, as illustrated in Figure 10b.

The parameters that can be modified in a honeycomb structure include the size of the cells and the thickness of the cell walls. The following experiments are designed to investigate the influence of geometric parameters (cell length, wall thickness) on structural displacement, strain field distribution, and output voltage. The objective of this systematic exploration is twofold: first, to reveal the underlying mechanism by which NPR structures enhance electromechanical conversion efficiency, and second, to validate their engineering applicability.

**Figure 11:**
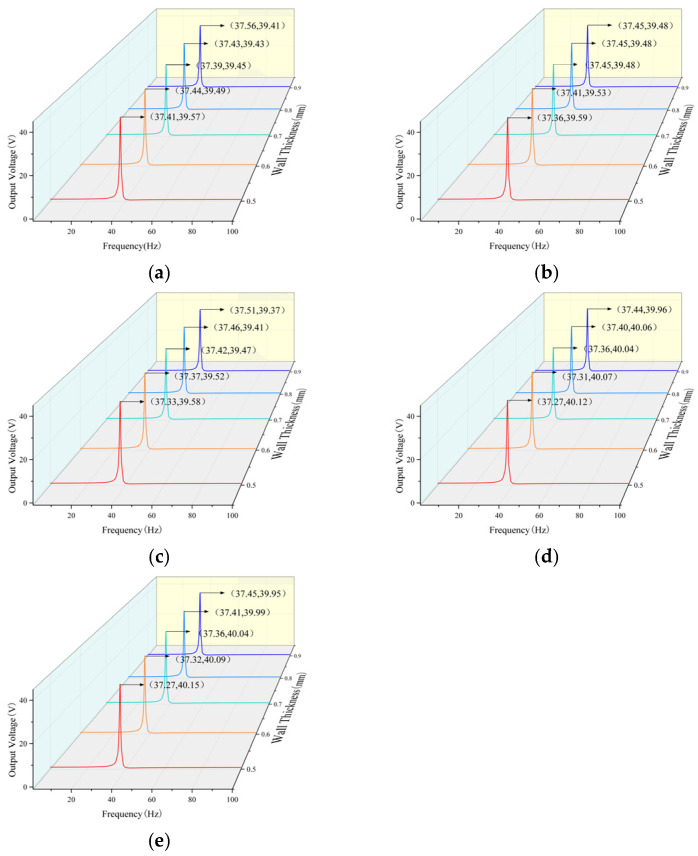
Simulation results graph of negative Poisson’s ratio cantilever beam parameters. (**a**) Cell length: 3 mm; (**b**) Cell length: 4 mm; (**c**) Cell length: 5 mm; (**d**) Cell length: 6 mm; (**e**) Cell length: 7 mm.

The present study sought to ascertain the impact of unit geometric dimensions on the system under investigation. To this end, the thickness of the honeycomb structure cells was systematically varied from 0.5 mm to 0.9 mm while maintaining a constant diagonal length. To this end, a simulation analysis was conducted to examine the first-order natural frequency and output voltage characteristics. The results of this analysis are presented in Figure 11.

An analysis of the data reveals that the first-order natural frequency and peak voltage of the curves in the image exceed those of the flat plate structure. Consequently, the hexagonal negative Poisson’s ratio cantilever beam structure exhibits a substantial optimization effect on the output voltage of the energy harvester. A comparative analysis of multiple diagrams reveals two distinct patterns. It has been demonstrated that, given a constant unit length, the first-order natural frequency increases in proportion to the increase in unit wall thickness. Conversely, the maximum output voltage decreases in proportion to the increase in unit wall thickness.

A comparative analysis of multiple diagrams reveals that, under constant unit wall thickness, the first-order natural frequency decreases while the output voltage increases with an increase in unit length. Specifically, at a unit length of 7 mm and a wall thickness of 0.5 mm, the energy harvester demonstrates its optimal output curve, exhibiting a first-order natural frequency of 37.27 hertz and an output voltage of 40.15 volts. Consequently, the implementation of a negative Poisson’s ratio configuration has been demonstrated to enhance the overall performance of cantilever beam piezoelectric energy harvesters, leading to a maximum output voltage increase of 1.06 V (2.7%) and a first-order natural frequency reduction of 1.63 Hz (4.1%). When identical input excitation is applied, the cantilever beam with a Poisson’s ratio structure optimizes strain distribution through its porous architecture, thereby delivering higher energy transfer efficiency for piezoelectric materials and enhancing charge density within the piezoelectric layer.

**Figure 12:**
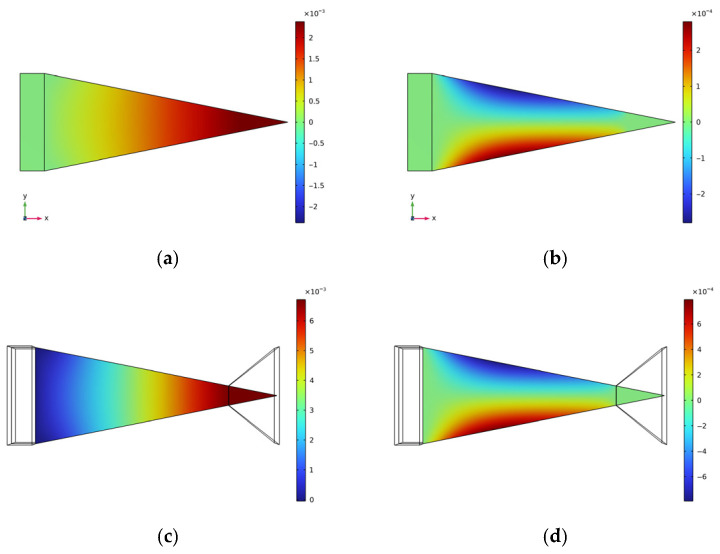
Flat plate structure displacement diagram. (**a**) Axial displacement of the cantilever beam substrate along the *X*-axis; (**b**) Axial displacement of the cantilever beam substrate along the *Y*-axis; (**c**) Piezoelectric sheet *X*-axis displacement; (**d**) Piezoelectric sheet *Y*-axis displacement.

**Figure 13:**
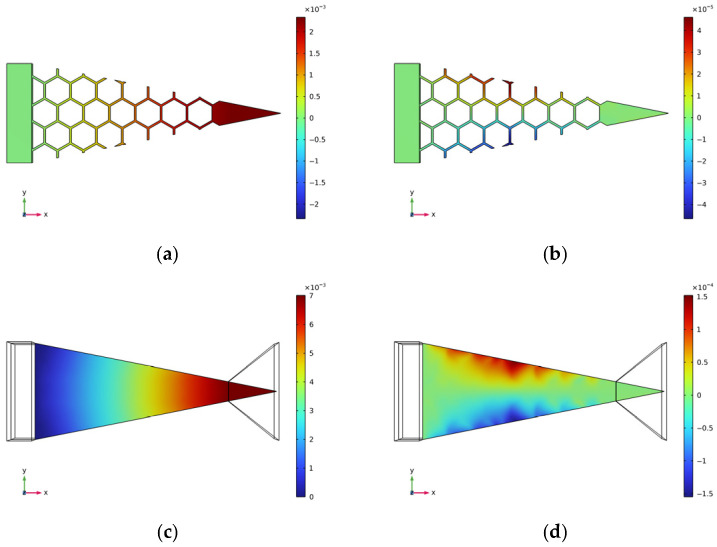
Negative Poisson’s ratio structural substrate displacement map. (**a**) Axial displacement of the cantilever beam substrate along the *X*-axis; (**b**) Axial displacement of the cantilever beam substrate along the *Y*-axis; (**c**) Piezoelectric sheet *X*-axis displacement; (**d**) Piezoelectric sheet *Y*-axis displacement.

Figure 12 and Figure 13, respectively, present the contour plots of displacement distribution for two cantilever beam structures under excitation at their respective natural frequencies, with measurements expressed in meters. The structural parameters employed for the model depicted in Figure 13 are as follows: a cell length of 7 mm and a wall thickness of 0.5 mm. This configuration is consistent with the optimal parameter structure previously delineated.

A thorough examination of Figure 12a,c and Figure 13a,c reveals that both cantilever beams and piezoelectric elements demonstrate positive displacement along the *X*-axis direction, thereby indicating that they are under tensile stress in this particular direction.

As demonstrated in Figure 12b,d, the displacements induced along the two edges of the cantilever beam in the flat plate structure are concentrated near the beam’s center, exhibiting contraction deformation and demonstrating a positive Poisson’s ratio effect.

Conversely, the behavior of structures with negative Poisson’s ratio is precisely the opposite. The edges of the beam are distant from the center of the cantilever, and the beam’s area increases, manifesting a negative Poisson’s ratio effect. When the cantilever beam substrate undergoes an axial (X-direction) tensile load, elongation deformation also occurs in the direction perpendicular to this load (Y-direction). Therefore, it can be concluded that the cantilever beam is also under tensile stress along the *Y*-axis.

This coupled deformation characteristic significantly alters the strain state of the piezoelectric layer, causing it to exhibit deformation along the *X*-axis and generate a substantial deformation component along the *Y*-axis. The enhancement in biaxial strain has been demonstrated to effectively augment the conversion efficiency of the piezoelectric effect, thereby leading to an improvement in the voltage output performance of the entire energy harvester.

### 3.3. Output Analysis of Variable-Density Mass Blocks

**Figure 14:**
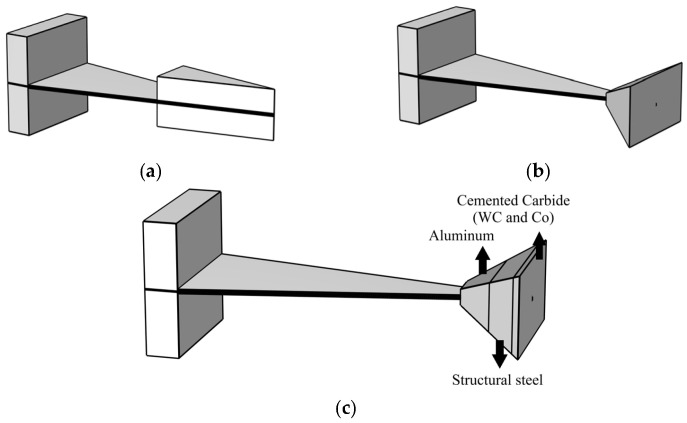
Schematic diagram of the energy harvester structure. (**a**) Structure I; (**b**) Structure II; (**c**) Structure III.

As shown in Figure 14a, this is a conventional energy harvester that has not undergone structural optimization. The structure incorporates a cantilever beam with a flat plate design.

As illustrated in Figure 14b, the energy harvester under consideration features a cantilever beam with a negative Poisson’s ratio structure, wherein the mass block consists exclusively of structural steel. The structure depicted in Figure 13b is analogous to that illustrated in Figure 10b in Section 3.2.

As illustrated in Figure 14c, the model incorporates non-uniform density mass blocks composed of aluminum- structural steel- cemented carbide, building upon the optimizations from the preceding two sections. Following a thorough calculation, it was ascertained that the weights of the mass blocks in all three structures remain constant. The cantilever beam substrates depicted in Figure 14b,c are composed of a honeycomb structure with optimal parameters, as delineated in Section 3.2 (i.e., Figure 12, with a cell length of 7 mm and a wall thickness of 0.5 mm).

**Figure 15:**
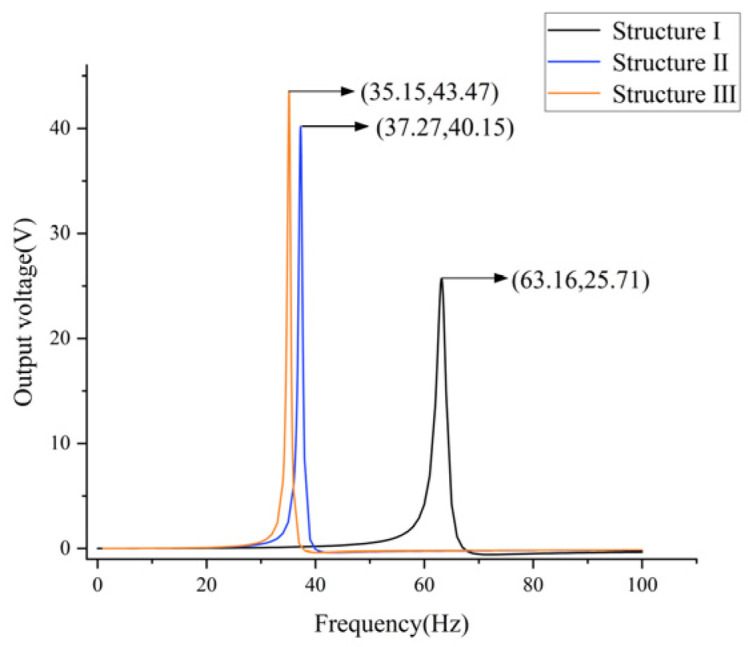
Output response curve graph.

As demonstrated in Figure 15, the optimized Structure Ⅲ demonstrates both the highest output voltage and the lowest natural frequency. A comparison of Structures Ⅱ and 3 reveals that the latter, featuring non-uniform density mass blocks, exhibits an increase in voltage of 3.32 V, representing an approximate 8.26% rise. Concurrently, the natural frequency of Structure Ⅲ experiences a decrease of 2.12 Hz, reflecting a 5.68% reduction. The findings indicate that a mass block with non-uniform density, composed of aluminum, structural steel, and cemented carbide, can substantially enhance the electromechanical performance of the energy absorber.

A comparison of Structures Ⅰ and Ⅲ reveals that the latter exhibits an output voltage increase of approximately 69.07% (a voltage rise of 17.76 V) and a natural frequency reduction of 44.34% (a frequency decrease of 28.01 Hz). In the context of multi-objective optimization, the performance of the energy harvester is enhanced. This enhancement is achieved through the integration of embedded coupling connections, negative Poisson’s ratio cantilever beams, and non-uniform mass density. The result of this integration is a synergistic improvement in both energy conversion efficiency and low-frequency adaptability.

## 4. Physical Experiment

**Figure 16:**
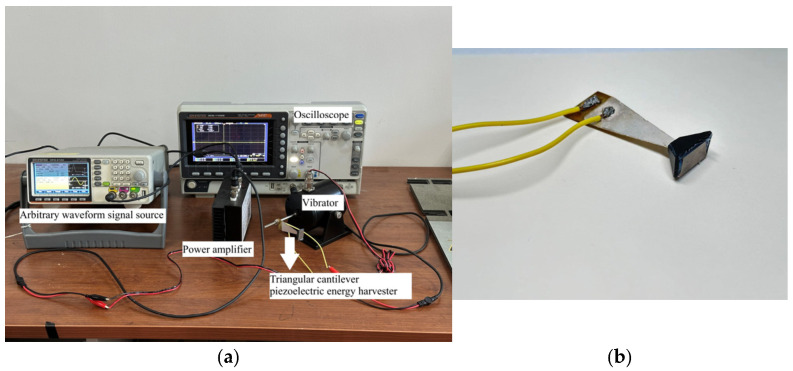
Photographs of Physics Experiments. (**a**) Experimental platform; (**b**) Prototype Close-Up Image.

In order to ascertain the precision of the finite element simulation results, an experimental platform was designed and constructed. This platform was based on the structural parameters of the piezoelectric energy harvester, as illustrated in Figure 16. An arbitrary waveform signal source is utilized to generate electrical signals of specific frequencies. The signal generator’s output is amplified by a power amplifier and fed to the vibrator, which receives the signal and generates vibrations. The vibrations induce deformations in the cantilever beam piezoelectric energy harvester. The piezoelectric elements are connected to the substrate via wires, with the other ends of the wires connected to an oscilloscope to capture the output voltage signals and display the voltage waveforms.

As illustrated in Figure 16b, a close-up view of the prototype is presented, with the model being derived from Figure 14c. The specific technical specifications of the cantilever beam piezoelectric energy harvester specimen utilized are as follows:

Cantilever Beam Substrate: Material: The dimensions of the brass component are as follows: maximum length × width × thickness = 55 × 20 × 0.2 mm. These structural dimensions and parameters are consistent with the finite element model. The parameters, including density and Young’s modulus, are derived from the material supplier’s data sheet and are consistent with the values reported in Table 1.

Piezoelectric Layer: The device utilizes PZT-5A piezoelectric ceramic disks (Yuan Doctor Technology, Guilin, China). When observed from a superior vantage point, the base measures 20 mm in diameter, the height reaches 50 mm, and the thickness is 0.2 mm. Wires extend from the cantilever beam substrate and piezoelectric element for the purpose of measuring voltage data. The material parameters are derived from the typical values provided by suppliers and are consistent with the values in Table 1.

Mass blocks: The fabrication of this component involved the use of aluminum, structural steel, and cemented carbide, as depicted in Figure 14c. Furthermore, a 1 mm thick acrylic plate is affixed to the surface to ensure structural stability. Preliminary calculations indicate that the combined weight and volume of the acrylic plate and epoxy resin are significantly less than those of the metal used to form the mass block. Consequently, the presence of the acrylic plate and epoxy resin is not expected to compromise the experimental results.

For excitation inputs, a fixed-frequency sinusoidal excitation was set via the signal source, with the experimental frequency range spanning from 1 Hz to 100 Hz. The objective of this study is to control the acceleration amplitude of the fundamental excitation. It is imperative to maintain the acceleration amplitude of the structural vibration at 1 m/s^2^ throughout the entire experimental frequency range. This is achieved by calculating the signal input to the vibrator.

For measurement instrumentation, the output voltage of the piezoelectric energy harvester is measured through a load resistor of known resistance, set at 12 kΩ to match the simulation parameters. The voltage across the resistor is measured using an oscilloscope (GOS-11028, GwinStek, Suzhou, China). The device under consideration provides a 100-megahertz bandwidth and a sampling rate of 1 gigahertz per second, which is considerably higher than the highest frequency of the signal.

**Figure 17:**
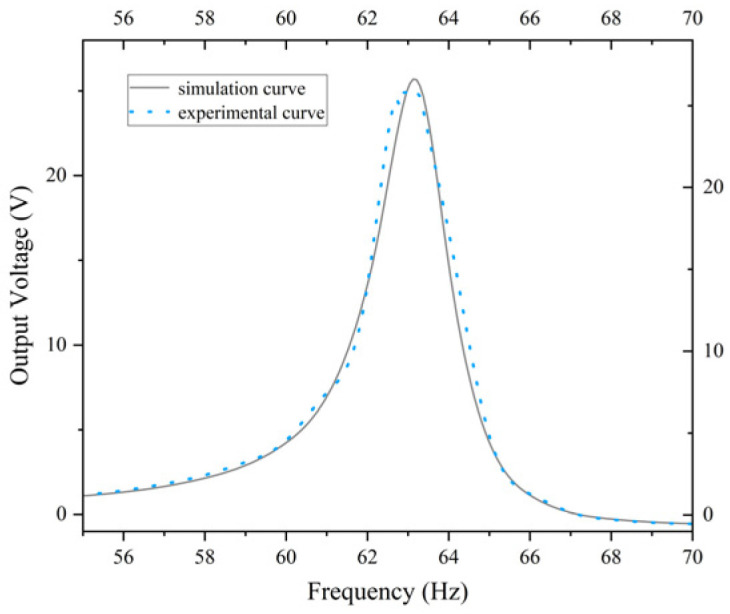
Simulation curve and physical experiment curve for the standard energy harvester.

**Figure 18:**
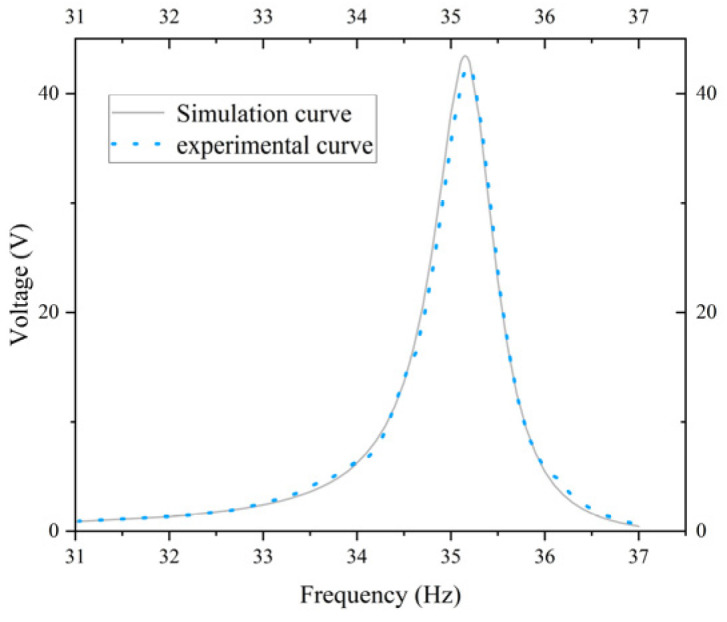
Simulation curve and physical experiment curve of the optimized energy harvester.

The objective of this study is to ascertain the validity of the theoretical model by comparing the voltage-frequency characteristic curves obtained from simulation and experiment. As illustrated in Figure 17, the simulated and experimental curves for the standard energy harvester constructed based on Figure 14a are presented. It is evident that the simulated curve exhibits a strong alignment with the experimental curve, with peak errors amounting to approximately 4.5%. As illustrated in Figure 18, the simulated and experimental curves of the optimized energy harvester constructed based on Figure 14c are presented. As shown in Figure 18, the two curves demonstrate a high degree of consistency within the 31–37 Hz frequency range, with both reaching a maximum near 35 Hz and subsequently decaying gradually. The relative error between voltage and frequency remains below 3%, indicating the model’s high accuracy in predicting system responses. The calculations indicate that the optimized energy harvester delivers a 71.26% increase in output voltage compared to the standard energy harvester. The simulation curve demonstrates a strong correlation with the experimental curve in terms of overall shape and peak characteristics, thereby substantiating the reliability of the theoretical model. This finding indicates that the model is capable of effectively reflecting the system’s overall dynamic behavior. The congruence between the simulation and experimental results substantiates the efficacy of the theoretical model developed, thereby establishing a foundation for its applicability in pertinent engineering applications.

**Figure 19:**
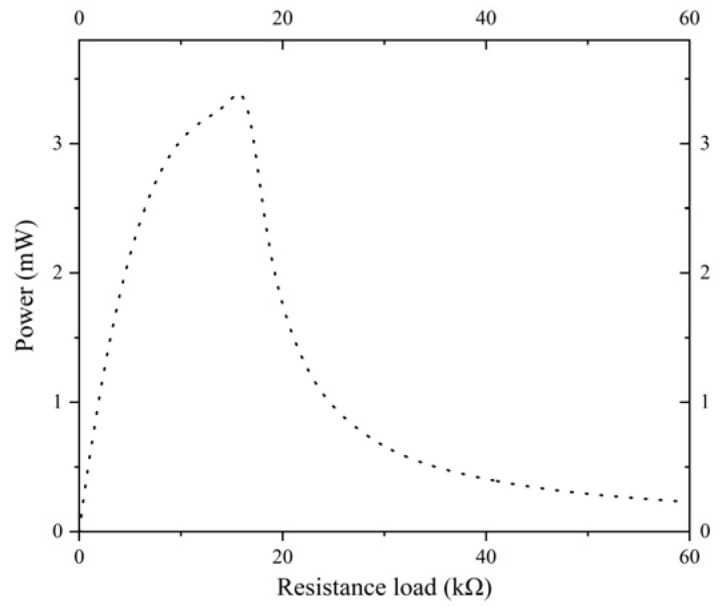
Power–Resistance load response curve.

This Figure 19 illustrates the characteristic curve of the energy harvester’s output power as a function of external load resistance. The horizontal axis of the coordinate system denotes load resistance, while the vertical axis denotes output power. The curve displays a conventional single-peak characteristic. The output power reaches its maximum value of 3.3 mW at a load resistance of approximately 16.4 kΩ.

## 5. Conclusions

This study addresses the limitations of conventional cantilever beam piezoelectric energy harvesters (PEHs) in terms of energy conversion efficiency by proposing a comprehensive structural optimization framework. The overarching objective of the proposed framework is to achieve a substantial enhancement in the electromechanical conversion performance of triangular cantilever beam PEHs through the meticulous morphological reconstruction of critical components. The study systematically optimized the connection method between the mass block and cantilever beam substrate, cantilever beam substrate structure, and the mass block material distribution through a multi-stage approach. The validation of the optimization results was conducted through finite element simulations and experiments.

The simulation results indicate that the voltage output increased by 69.07% (a rise of 17.76 V), while the resonance frequency decreased by 44.34% (a reduction of 28.01 Hz). Specifically, the embedded coupling connection increased voltage by 37.06% and reduced frequency by 34.81%. Structural optimization of the mass block boosted voltage by 10.9% and lowered frequency by 6.9%. The NPR substrate further improved strain uniformity, raising voltage by 2.7%. Lastly, the variable-density mass block contributed an additional 18.41% voltage gain. These results substantiate the efficacy of structural optimization in enhancing stress distribution and piezoelectric effects.

The experimental verification section of the study involved the use of a physical test platform to obtain actual measurement data for the prototype. According to the experimental results, the optimized energy harvester demonstrated a 71.26% increase in output voltage compared to the standard energy harvester. The voltage-frequency characteristic curve of the optimized energy harvester exhibited a high degree of consistency within the 31–37 Hz range, with a relative error of less than 3%, thereby substantiating the reliability of the simulation model. Furthermore, the output power of the optimized energy harvester exhibited attains a maximum of 3.3 mW at a load resistance of 16.4 kΩ, thereby substantiating the viability of the optimized structure in practical applications.

This study makes a significant contribution by proposing a scalable structural optimization framework that offers a novel approach for designing high-performance, low-frequency adaptive PEHs. These are suitable for microelectromechanical systems (MEMS) and embedded energy applications.

## Figures and Tables

**Table 1:** Material Parameters Table.

Assembly Unit	Parameter	Value
Structural steel	density	7850 kg/m^3^
	Young’s modulus	200 × 10^9^ Pa
Copper	density	8960 kg/m^3^
	Young’s modulus	120 × 10^9^ Pa
Aluminum	density	2700 kg/m^3^
	Young’s modulus	70 × 10^9^ Pa
Cemented Carbide (WC and Co) Piezoelectric ceramic PZT-5A	density	14,461 kg/m^3^
	Young’s modulus	570 × 10^9^ Pa
	density	7850 kg/m^3^
	Young’s modulus	200 × 10^9^ Pa
	piezoelectric strain constant	(185, 400, 640, 9.6, 25.6)
	relative dielectric constant	(919.1, 919.1, 826.6)

## Data Availability

The original contributions presented in this study are included in the article. Further inquiries can be directed to the corresponding author.

## Abbreviations

The following abbreviations are used in this manuscript:

PEHs	piezoelectric energy harvesters
IoT	Internet of Things
MEMS	Microelectromechanical systems
NPR	Negative Poisson’s ratio
